# Humidity Sensors Based on Metal–Organic Frameworks

**DOI:** 10.3390/nano12234208

**Published:** 2022-11-26

**Authors:** Ke Wu, Teng Fei, Tong Zhang

**Affiliations:** State Key Laboratory of Integrated Optoelectronics, College of Electronic Science and Engineering, Jilin University, Changchun 130012, China

**Keywords:** metal−organic frameworks, humidity sensor, sensing materials, sensing mechanism, impedance, QCM, fluorescent

## Abstract

Humidity sensors are important in industrial fields and human activities. Metal−organic frameworks (MOFs) and their derivatives are a class of promising humidity−sensing materials with the characteristics of a large specific surface area, high porosity, modifiable frameworks, and high stability. The drawbacks of MOFs, such as poor film formation, low electrical conductivity, and limited hydrophilicity, have been gradually overcome with the development of material science. Currently, it is moving towards a critical development stage of MOF−based humidity sensors from usability to ease of use, of which great challenges remain unsolved. In order to better understand the related challenges and point out the direction for the future development of MOF−based humidity sensors, we reviewed the development of such sensors based on related published work, focusing on six primary types (impedance, capacitive, resistive, fluorescent, quartz crystal microbalance (QCM), and others) and analyzed the sensing mechanism, material design, and sensing performance involved, and presented our thoughts on the possible future research directions.

## 1. Introduction

Humidity sensors play an important role in human health [1,2,3,4], electronic component factories [5,6,7], grain storage [8,9,10], etc. To achieve a more accurate and wider range of humidity detectors, sensing materials that are more sensitive to water molecules are urgently required. In addition, humidity−sensing properties such as response, sensitivity, linearity, hysteresis, response time, and long−term stability are highly related to the characteristics of sensing materials [11,12,13,14]. Normally, humidity−sensing materials are hydrophilic, porous, and stable [15]. Until now, humidity sensors based on different types of sensing materials such as metal oxides [12], polymers [16,17,18,19,20], and carbon materials [21,22] have been reported. Metal−organic frameworks (MOFs), types of compounds famous for their characteristics of porosity, adjustable structure, and good chemical stability [23,24,25], have been widely applied in the fields of gas separation and storage [26,27], catalysis [28,29], ion batteries [30,31], etc. The advantages of porosity and an adjustable structure make MOFs promising materials in chemical sensing [32,33,34,35]. Until now, sensors based on MOFs and their derivatives have been applied in gas sensing [36,37], nitro explosive detection [38,39,40,41], antibiotic detection [42,43], etc.

In recent years, MOF−based humidity sensors have received more and more attention [44]. The main mechanism of MOF gas sensors is based on the interaction between MOFs and the target gas [45], while the main point of humidity sensors is the adsorption of water molecules [46,47]. There are many reviews on MOF−based gas sensors [48,49], although none of them focus on the application of MOFs in humidity sensors. In this review, firstly, we summarize the sensing mechanisms of different types of MOF−based humidity sensors. Secondly, we review the development of different types of MOF−based humidity sensors in terms of MOF selection and design principle as well as sensing performance. Finally, we point out encouraging application prospects and present our thoughts on the possible future research directions.

## 2. Humidity Sensors Based on MOFs

**Figure 1:**
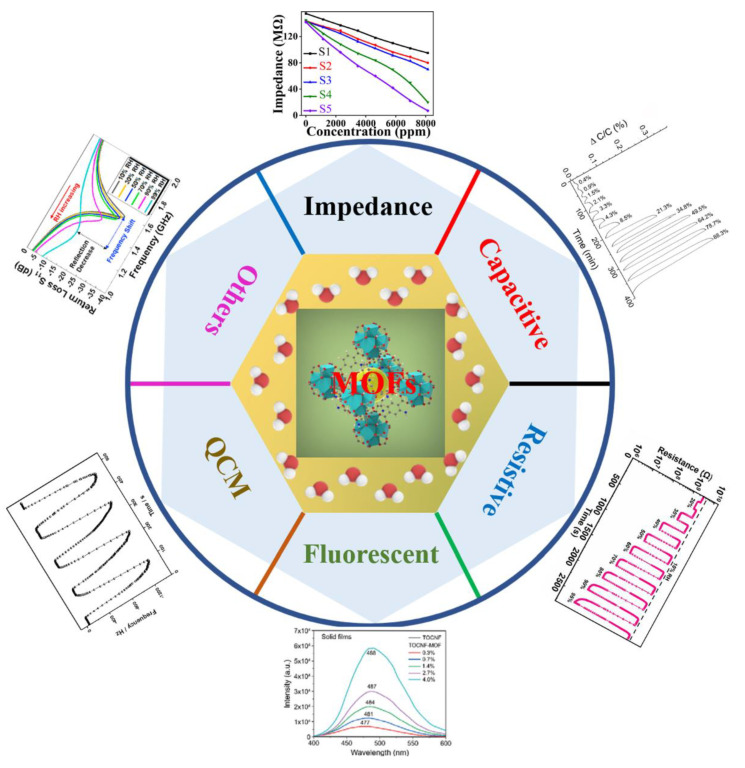
Different types of MOF−based humidity sensors. Reproduced with permission from Ref. [53]. Copyright 2022, IEEE. Reproduced with permission from Ref. [54]. Copyright 2020, American Chemical Society. Reproduced with permission from Ref. [55]. Copyright 2022, Elsevier. Reproduced with permission from Ref. [56]. Copyright 2022, Wiley−VCH GmbH. Reproduced with permission from Ref. [57]. Copyright 2017, American Chemical Society. Reproduced with permission from Ref. [58]. Copyright 2021, Elsevier.

The structural characteristics of MOFs make them suitable for humidity sensing. Firstly, MOFs own a large specific surface area and high porosity [50,51]. The interaction between the sensing materials and water molecules would affect the humidity−sensing properties, and a larger specific surface area and high porosity could expose more active sites [1]. In addition, pores or channels are known as the pathway for transporting water molecules [15]. Therefore, MOFs with a large specific surface area and high porosity are suitable for humidity sensing. Secondly, the structures of MOFs are adjustable [52]. The surface characteristic of the material is important in chemical sensing [8], and the adjustable structure of MOFs endows the MOF−based humidity sensors with adjustable hydrophilicity. Additionally, it is also important to determine the water adsorption capacity of a humidity sensor. Different methods can be used to measure the water adsorption capacity of MOF−based humidity sensors, for example, using QCM sensors or measuring water sorption isotherms. By combining the measurable water adsorption capacity and adjustable structure, high−performance MOF−based humidity sensors can be obtained. Thirdly, MOFs are stable. Humidity sensors that can work effectively for long periods of time are welcome, which requires the sensing materials to be highly stable [15]. The chemical bonds between the metal centers and the oxygen and nitrogen in the linkers endow the MOFs with good chemical stability, which is conducive to the preparation of highly stable MOF−based humidity sensors. Humidity sensors based on MOFs or their derivatives can be divided into various types following different output signals (Figure 1).

**Figure 2:**
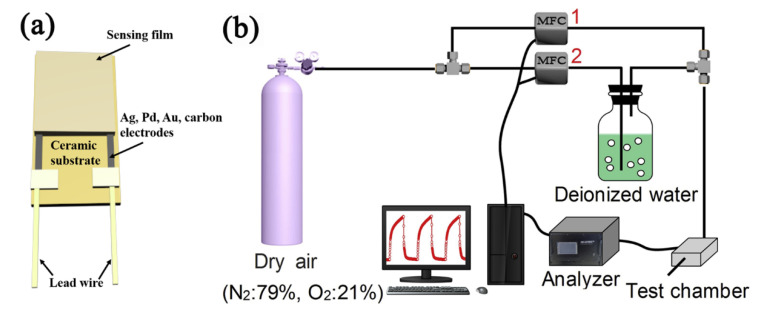
Schematic diagram of (**a**) ceramic−based interdigitated electrode. (**b**) Dynamic air distribution system and humidity sensing testing system. Reproduced with permission from Ref. [21]. Copyright 2022, Elsevier.

The working process of humidity sensors includes the adsorption of water molecules, signal conversion, signal transmission, and signal output, in which the adsorption of water molecules and the signal conversion and transmission process occurs in the sensing materials [59]. When the sensing materials adsorb water molecules, their physical properties will change, and the changed physical properties are then converted into measurable signals, including fluorescence intensity, frequency, impedance, resistance, and capacitance. The hydrophilicity of the substrate, the number of electrodes, and the electrode material show various effects on the signals [60]. As an example, electrodes of electrically transduced humidity sensors are usually prepared on the substrates with the interdigital structure (Figure 2a). The collected signals are processed by subsequent data processing to determine the corresponding relationship between the signals and the humidity.

A humidity atmosphere−providing system and a signal−receiving system are equally important for researching humidity sensors. Humidity atmospheres can be provided by saturated salt solutions [5], but the humidity interval is large. Dynamic humidity generators and dynamic air distribution systems can solve this problem (Figure 2b). More precise and wider humidity range atmospheres could be realized through the dynamic air distribution system and dynamic humidity generator. In addition, the reception of sensor signals mainly needs impedance analyzers, electrochemical workstations, fluorescence analyzers, frequency analyzers, etc.

**Figure 3:**
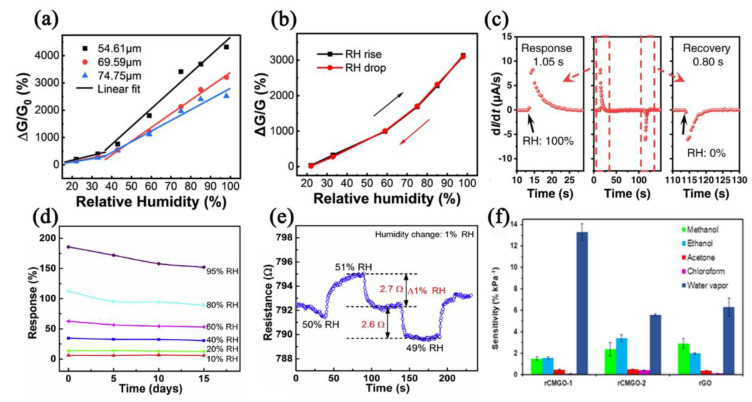
(**a**) Schematic diagram of response curves. (**b**) Schematic diagram of hysteresis curves. Reproduced with permission from Ref. [17]. Copyright 2022, Springer Nature. (**c**) Schematic diagram of response/recovery curve and corresponding response time and recovery time. Reproduced with permission from Ref. [61]. Copyright 2022, Springer Nature. (**d**) Schematic diagram of impedance values vs. time. (**e**) Schematic diagram of response curve of a humidity sensor to 1% RH change (resolution). Reproduced with permission from Ref. [21]. Copyright 2021, Elsevier. (**f**) Schematic diagram of selectivity of a humidity sensor to different gas vapor. Reproduced with permission from Ref. [66]. Copyright 2014, American Chemical Society.

For practical applications, different parameters of a humidity sensor should be considered, including response, sensitivity, hysteresis, linearity, response/recovery time, selectivity, the limit of detection (LOD), and long−term stability (take electrically transduced humidity sensors as an example, Figure 3). The response is defined as the signal value change of the humidity sensors obtained between a humid atmosphere and a dry atmosphere [61]. The response can be expressed by Equation (1) (take an impedance−type humidity sensor as an example).

Response = *Z_dry_*/*Z_wet_* (1)

where *Z_dry_* represents the impedance value of sensors in a dry atmosphere and *Z_wet_* represents the impedance value of sensors in a wet atmosphere. The sensitivity is defined as the ratio of the response and water concentration (response/water concentration). However, in some reported work, there is no strict distinction between response and sensitivity. The working range is defined as the humidity range within which humidity sensors can work normally (without affecting the accuracy and stability). Different working ranges correspond to different application scenarios [62]. Hysteresis is defined as the maximum misalignment of the abscissa between the adsorption curve and the desorption curve. The value of hysteresis is directly related to the accuracy and repeatability of a humidity sensor [63]. The response and recovery time is defined as the time needed for the signals of the humidity sensors to reach 90% of the variation [64]. If the signal of a humidity sensor fails to respond or recover to 90% of its original value in a continuous response/recovery curve, it can be considered that the humidity sensor does not have repeatability, which can also be called an unrecoverable sensor or an unstable sensor [49]. Long−term stability is used to describe the remaining sensing performance of humidity sensors after continuous operation for a period of time and is a key factor that indicates the reliability and stability of a humidity sensor [65], which provides key information for the research of sensor aging and sensor calibration. The resolution is defined as the smallest difference of water molecule concentration that a humidity sensor can distinguish [21]. A small resolution means highly accurate identification.

### 2.1. Electrically Transduced Humidity Sensors

#### 2.1.1. Basic Sensing Principles of Electrically Transduced Humidity Sensors

An electrically transduced humidity sensor consists of a sensing layer, an electrode, and a signal collection system [67]. The sensing layer needs to realize the adsorption, interaction, and transport of water molecules as well as the change and transport of electrical parameters [68]. For the adsorption of water molecules, sensing materials need to be hydrophilic; for the transport of water molecules, pathways are needed [69]; and for electrical parameter changes and transmission, good semiconductor properties and a good connection between the sensing film and the electrode are required [70,71]. The sensing layer usually includes three types: powder pellet, high−quality thin/thick film, and single crystal [66,72,73,74]. Generally speaking, a sensing layer with high−quality film is the most common choice for researchers [75].

#### 2.1.2. MOF−Based Impedance−Type Humidity Sensors

Impedance−type humidity sensors have been extensively researched due to their low cost, miniaturization, and user friendliness. Considering the structural advantages of MOFs, MOF−based impedance−type humidity sensors have been widely investigated (Table 1).

In 2009, Achmann et al. [76] investigated the electrically transduced humidity−sensing properties of MOFs for the first time. Under the test frequency of 1 Hz, the Fe−BTC sensor and Al−BTC sensor showed a decrease in the impedance when the water concentration increased from 0 vol% H_2_O to 2.5 vol% H_2_O, and the sensitivity was 30.4 MΩ/vol% H_2_O and 590 MΩ/vol% H_2_O at 120℃, respectively. Moreover, the Fe−BTC sensor can fully recover to the initial impedance value in a dry environment. This work demonstrates the possibility of using MOFs as sensitive materials to prepare recyclable humidity sensors, but the high working temperature makes the prepared sensor unsuitable for wide−ranging applications.

**Figure 4:**
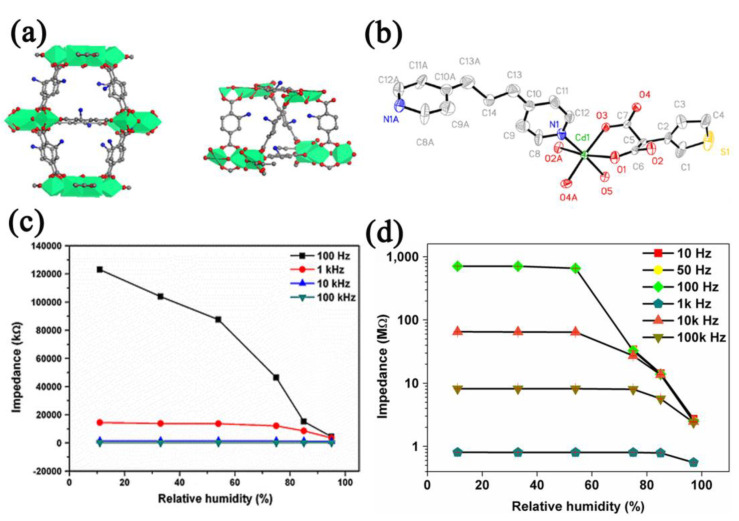
(**a**) Crystal structure of NH_2_−MIL−125(Ti). Reproduced with permission from Ref. [77]. Copyright 2013, Springer Nature. (**b**) Coordination environment of [Cd(TMA)(DPP)_0_._5_∙H_2_O]*_n_*. Reproduced with permission from Ref. [78]. Copyright 2018, Elsevier B.V. (**c**) Response curves vs. frequencies of NH_2_−MIL−125(Ti)−based humidity sensor in the humidity range of 11–95% RH. Reproduced with permission from Ref. [77]. Copyright 2013, Springer Nature. (**d**) Response curves vs. frequencies of [Cd(TMA)(DPP)_0_._5_∙H_2_O]*_n_*−based humidity sensor in the humidity range of 11–95% RH. Reproduced with permission from Ref. [78]. Copyright 2018, Elsevier.

The hydrophilicity of the sensing materials is important for humidity sensing. The introduction of hydrophilic groups can improve the hydrophilicity of MOFs [89,90]. For example, Zhang et al. [77] prepared NH_2_−MIL−125(Ti) with high porosity and good mechanical properties. Since metal−oxygen groups (Ti−O) and amino groups (−NH_2_) are hydrophilic (Figure 4a), the NH_2_−MIL−125(Ti)−based sensor showed a decrease in the impedance as the humidity increased (11–95% RH, response: 27.5, Figure 4c) with a hysteresis of ~5% RH, and the response time and recovery time were calculated to be 45 s and 50 s, respectively. Yin et al. [78] prepared a three−dimensional (3D) MOF [Cd(TMA)(DPP)_0_._5_∙H_2_O]*_n_* (H_2_TMA = 3−thiophenemalonic acid, DPP = 1,3−di(4−pyridyl)propane) with O, N, and uncoordinated S atoms (Figure 4b). The hydrogen bonds between the O, N, and S atoms and water molecules make the MOF hydrophilic, and the MOF−based sensor showed a response of ~350 (Figure 4d), a hysteresis of <2% RH, and a response time of 11 s in the humidity range of 11–97% RH. Zhang et al. [79] researched the sensing performance of a MIL−101(Cr) −based humidity sensor. Benefiting from the strong hydrophilicity of metal−oxygen clusters (Cr−O), the sensor showed >1900 times the impedance change in the humidity range of 33–95% RH, with a small humidity hysteresis (4% RH) and a fast response (17 s).

Hong Kong University of Science and Technology−1 (HKUST−1), a copper−based MOF with uncoordinated metal sites and hydrophilic ligands, is highly hydrophilic [91]. Wang et al. [80] prepared HKUST−1 with two kinds of morphologies (ultrathin nanosheets and octahedral structures) and researched their humidity−sensing properties. The HKUST−1 nanosheet−based sensor showed a higher response (>100) and a shorter response time of 2 s than the octahedral HKUST−1−based sensor (response: ~10, response time: 11 s). It was considered that, compared to an octahedron structure, a nanosheet structure could expose more active sites and thus have a better hygroscopic effect.

**Figure 5:**
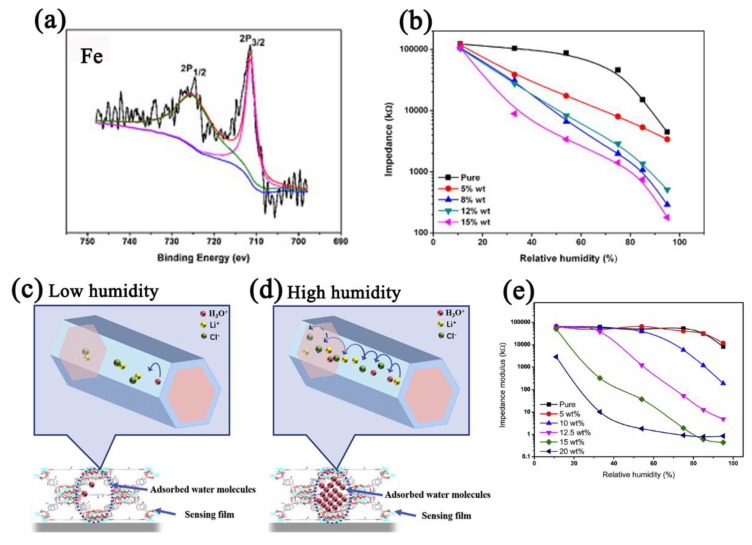
(**a**) XPS pattern of FeCl_3_−NH_2_−MIL−125(Ti). (**b**) Response curves of FeCl_3_−NH_2_−MIL−125(Ti)−based humidity sensors to humidity in the range of 11–95% RH. Reproduced with permission from Ref. [81]. Copyright 2014, Elsevier. Schematic of conduction path of LiCl@UiO−66−NH_2_−based humidity sensor in (**c**) low humidity and (**d**) high humidity. (**e**) Response curves of LiCl@UiO−66−NH_2_−based humidity sensors to humidity in the range of 11–95% RH. Reproduced with permission from Ref. [53]. Copyright 2020, Elsevier.

In addition to introducing hydrophilic ligands into MOFs, humidity−active materials could be directly loaded into the MOFs to obtain better humidity−sensing properties. Zhang et al. [81] prepared FeCl_3_−NH_2_−MIL−125(Ti) composites for humidity sensing. Due to the introduction of FeCl_3_ (Figure 5a), the hydrophilicity of the NH_2_−MIL−125(Ti) was enhanced, and the response of the FeCl_3_−NH_2_−MIL−125(Ti) sensor was ~367 in the humidity range of 11–95% RH (27.5 for NH_2_−MIL−125(Ti)−based sensor) (Figure 5b), and the response time and recovery time were calculated to be 11 s and 86 s, respectively. Zhang et al. [53] reported a LiCl@UiO−66−NH_2_−based humidity sensor. The strong hydrophilicity of LiCl makes the LiCl@UiO−66−NH_2_−based sensor highly sensitive to humidity changes (11–95% RH), with a high sensitivity (10^4^) (Figure 5c–e), a fast response (6 s), and good stability (>30 days). Su et al. [82] fabricated flexible humidity sensors by coating KOH/M050 and AuNPs/M050 on the PET substrate. KOH and Au NPs make the sensing films hydrophilic and reduce the initial impedance of the sensors. The KOH/M050−based sensor can work in 20–90% RH with a small humidity hysteresis (<2% RH).

**Figure 6:**
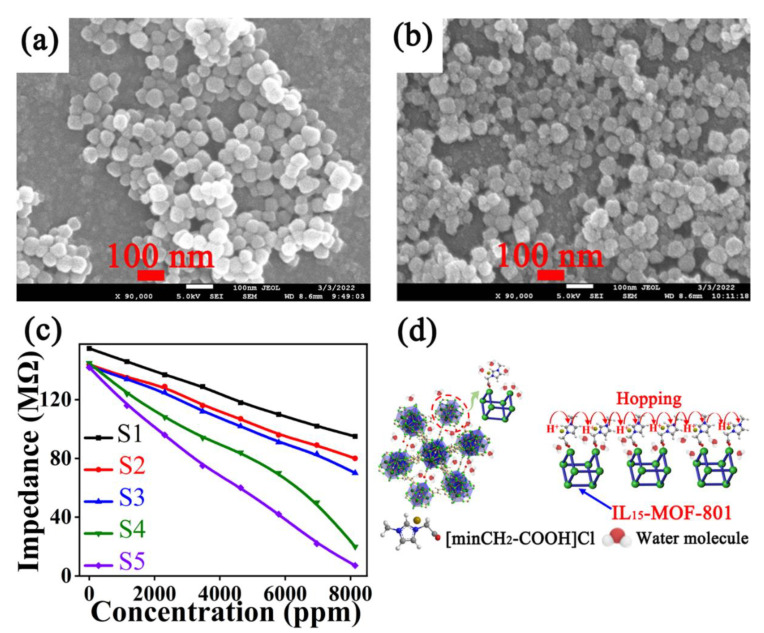
(**a**) SEM image of MOF−801 (scale: 100 nm). (**b**) SEM image of IL_15_−MOF−801 (scale: 100 nm). (**c**) Response curves of IL_x_−MOF−801−based humidity sensors to humidity in the range of 0–8138 ppmv. (**d**) Crystal structure and humidity−sensing mechanism of IL_15_−MOF−801−based humidity sensor. Reproduced with permission from Ref. [84]. Copyright 2022, IEEE.

In the abovementioned humidity sensors, the sensing films were mainly composed of MOFs powders; however, the poor film formability of MOF powders hinders the transmission of electrical signals and limits the improvement of the sensing performance. A high−quality sensing film is beneficial to the conversion and transmission of electrical signals. In order to obtain high−quality sensing film, it is necessary to control the particle size of the MOFs to the nanoscale. Cui et al. [83] synthesized Zn^2+^−modified UiO−66−NH_2_ with a particle size of ~200 nm, and the sensing film was prepared by drop coating. The humidity sensor with stable sensing film showed a high response (8070), a small hysteresis (1.81% RH), and a short response time (2 s). Wu et al. [84] prepared IL_x_−MOF−801 nanoparticles and the particle size was controlled to ~60 nm (Figure 6a,b). The sensing films were prepared by drop coating. MOF−801 is hydrophilic, and the introduced IL further enhanced the hydrophilicity and conductivity of MOF−801 (Figure 6c,d), so that the optimized IL_x_−MOF−801 sensor showed high sensitivity to low humidity (0–35% RH, 0–8138 ppmv), with a small hysteresis (1% RH), a fast response (400 ms), a high resolution (1% RH), and excellent long−term stability (>100 days).

**Figure 7:**
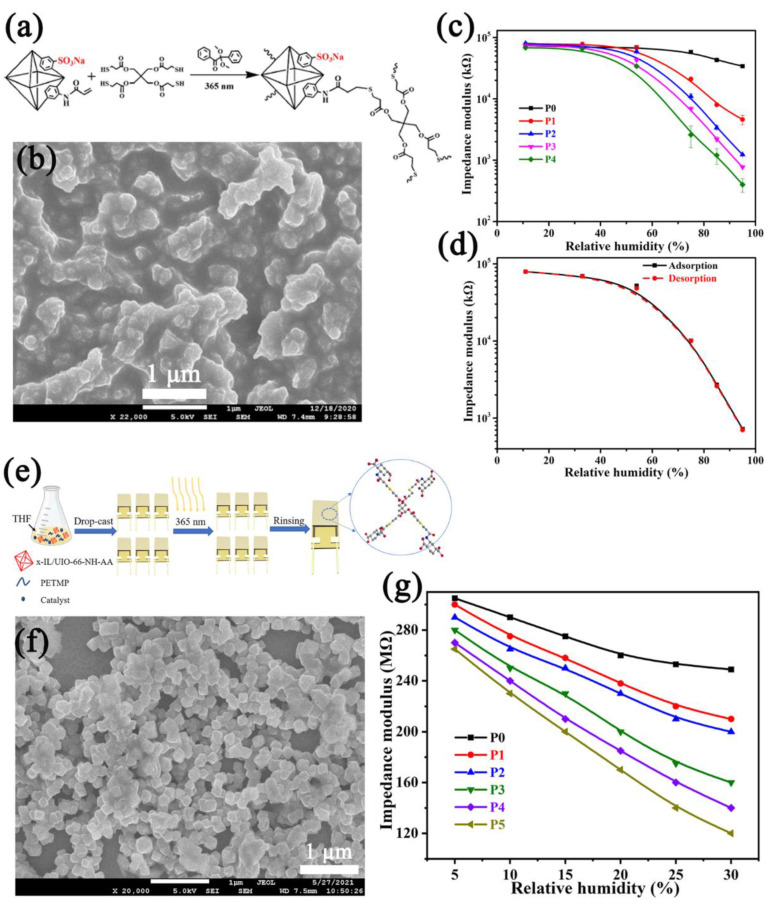
(**a**) Synthetic schematic diagram of alkene−thiol click reaction. (**b**) SEM image of MOF film after click reaction (scale: 1 μm). (**c**) Response curves of MOF film−based humidity sensors to humidity in the range of 11–95% RH. (**d**) Hysteresis curve of the optimized MOF film−based humidity sensor. Reproduced with permission from Ref. [85]. Copyright 2021, Elsevier B.V. (**e**) Flow chart for preparing MOF−based humidity sensors in batch through in situ click reaction on electrodes. (**f**) SEM image of IL−MOF polyelectrolyte film after click reaction (scale: 1 μm). (**g**) Response curves of IL−MOFs polyelectrolyte film−based humidity sensors to low humidity in the range of 5–30% RH. Reproduced with permission from Ref. [86]. Copyright 2022, Elsevier.

Sensing films prepared in situ on electrodes can solve the problems of the non-uniformity of the sensing layer and the poor contact with electrodes. Wu et al. [85] prepared MOF-based sensing films in situ on interdigital electrodes through alkene-thiol click reaction in batch (Figure 7a). The SEM images showed that the MOF formed continuous and dense film after the click reaction (Figure 7b), which is beneficial to the transmission of electrical signals and improves the parallelism. The optimized humidity sensor showed good sensing performance in the range of 11–95% RH with a hysteresis of 1.2% RH (Figure 7c,d), and because of the structural defects, the response time and recovery time reached 3.1 s and 1.5 s, respectively. They reported another humidity sensor with a similar method based on the UiO−66−NH_2_−derivative polyelectrolytes [86]. Parts of the ligands in UiO−66−NH_2_ were replaced by hydrophilic ionic liquid [minCH_2_−COOH]Cl, and the sensing films were prepared on the electrodes in situ with click reaction (Figure 7e,f). The optimized IL−MOF polyelectrolyte−based humidity sensor realized a low humidity−detection ability in the humidity range of 5–30% RH with a high response (~220%) (Figure 7g), a small hysteresis (0.2% RH), and a short response time (2.7 s).

As well as in situ film formation on electrodes, mixing MOFs as fillers in polymers is also a good method to form high−quality sensing films. Ru et al. [87] reported a thin−film humidity sensor with MIL−101 as the filler in hydrophilic sulfonated poly(ether ether ketone) (SPEEK). When the hydrophilic MOFs were filled into the SPEEK, the surface of the polymer became rough and porous, which is beneficial for water adsorption and desorption. The sensor exhibited a response of ~10^6^, a hysteresis of ~2% RH, and a fast response (9 s).

**Figure 8:**
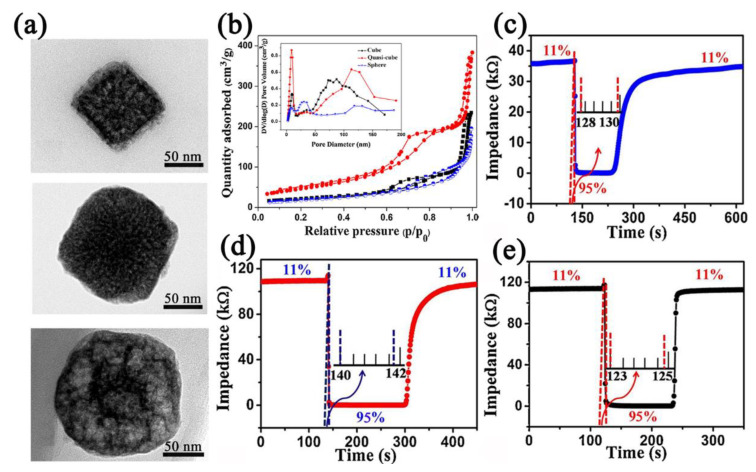
(**a**) TEM images of PB−derived Fe_2_O_3_ nanostructures with different morphologies. (**b**) N_2_ adsorption−desorption isotherms and corresponding pore size of different morphologies of Fe_2_O_3_ nanostructures. Response and recovery curves of different morphologies of Fe_2_O_3_−based humidity sensors between 11% RH and 95% RH (**c**) cubes, (**d**) quasi−cubes, and (**e**) spheres. Reproduced with permission from Ref. [88]. Copyright 2019, Elsevier.

Inspired by the excellent properties of MOFs’ derivatives in other sensing fields [49], Yu et al. [88] prepared the Prussian blue (PB) derivative Fe_2_O_3_ with different morphologies and researched the humidity−sensing properties (Figure 8a). The prepared Fe_2_O_3_ with different morphologies showed different porosities (Figure 8b). Among them, the Fe_2_O_3_−based humidity sensor with the largest specific surface area exhibited the highest response (1568) in 11–95% RH with a small hysteresis (0.59% RH) and a fast response (<2 s) (Figure 8c–e).

#### 2.1.3. MOF−Based Capacitive−Type Humidity Sensors

Capacitive−type humidity sensors are another attractive class of sensors [21], where the capacitance changes due to the change in the dielectric permittivity of sensing materials upon adsorbing water molecules. The dielectric permittivity of water is ~78.5 F/m (25 °C), while the dielectric permittivity of sensing materials is normally much smaller. The formula for calculating the capacitance of humidity sensors can be described by Equation (2) [21].

(2) $$C=\frac{\varepsilon S}{4\pi kd}$$

where *C* represents the capacitance of the humidity sensors, ε is the dielectric permittivity of the sensing materials, *S* and *d* are the area and distance between the parallel plates, respectively, and *k* is the electrostatic force constant (9.0 × 10^9^ N·m^2^/C^2^).

When water molecules are adsorbed on the sensing materials, the dielectric permittivity of the materials becomes larger, resulting in the increased capacitance of the sensors. The most important factor is the water adsorption ability, and the testing frequency also influences the capacitance [53]. Thanks to their adjustable water adsorption capacity, MOFs are suitable for fabricating high−performance capacitive−type humidity sensors (Table 2).

Achmann et al. [76] reported two MOFs (Fe−BTC and Al−BTC) for humidity sensing. When the water concentration increased from 0 vol% H_2_O to 2.3 vol% H_2_O, the capacitance of the Fe−BTC sensor showed an increase of 4.4 pF. Liu et al. [92] prepared a NH_2_−MIL−125(Ti)−based capacitive humidity sensor. The capacitance of the sensor showed an increase of 5397% with good linearity (R^2^ = 0.995), fast recovery (5 s), and a small hysteresis (~1.9% RH) in the humidity range of 11–75% RH. The high response contributed to the high surface area and highly hydrophilic characteristics of NH_2_−MIL−125(Ti). Seco et al. [93] reported a humidity sensor based on a 3D MOF [K_8_(ptca)_3_(H_3_O)_4_]*_n_* (ptca: perylene−3,4,9,10−tetracarboxylate). The capacitance of the sensor increased for more than five orders of magnitude when the humidity was higher than 40% RH, and showed no response to the temperature.

**Figure 9:**
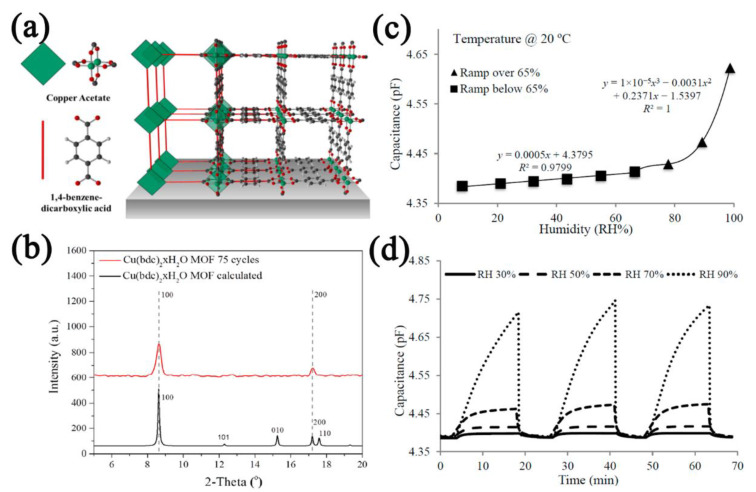
(**a**) Crystal structure, copper acetate, and organic linker of 2D Cu(bdc)·xH_2_O film. (**b**) XRD patterns of Cu(bdc)·xH_2_O film after 75 cycles and simulated. (**c**) Response curve of Cu(bdc)·xH_2_O−based sensor to humidity in the range of 11–100% RH. (**d**) Cycling curves of Cu(bdc)·xH_2_O−based sensor. Reproduced with permission from Ref. [95]. Copyright 1996–2022, MDPI, Basel, Switzerland.

Copper−based MOFs with strong hydrophilicity due to the presence of uncoordinated active sites are conducive to the preparation of capacitive−type humidity sensors. Liu et al. [94] prepared continuous Cu_3_(BTC)_2_ film through the method of homogeneous nucleation. The hydrophilic linker and copper metallic oxygen clusters make Cu_3_(BTC)_2_ highly hydrophilic. The capacitance of the Cu_3_(BTC)_2_−based sensor increased from 130.6 pF to 253.6 pF with a hysteresis of <1% RH and a sensitivity of ~1.499 pF/% RH in the humidity range of 11.3–84.3% RH. Sapsanis’s group [95] prepared continuous Cu(bdc)·xH_2_O film on interdigitated electrodes (IDEs) following the liquid−phase epitaxy method (Figure 9a,b). Cu(bdc)·xH_2_O owns open metal sites and thus has a strong adsorption capacity to water molecules. The sensor exhibited two capacitance change trends (below or over 65% RH), which were caused by the different ways that Cu(bdc)·xH_2_O interacted with the water molecules (Figure 9c,d). When the humidity was lower than 65% RH, the open copper metal sites played a key role, and when the humidity was higher than 65% RH, the hydrogen bonds between the water molecules dominated. Hosseini et al. [96] prepared continuous Cu−BTC film on copper electrodes using the electrochemical method and used it for the detection of very low humidity. The sensitivity of the Cu−BTC−based sensor in the humidity range of 20–100 ppm was 1.13 pF. Most importantly, the LOD was as low as 5.45 ppm, which is very rare for MOF−based humidity sensors.

**Figure 10:**
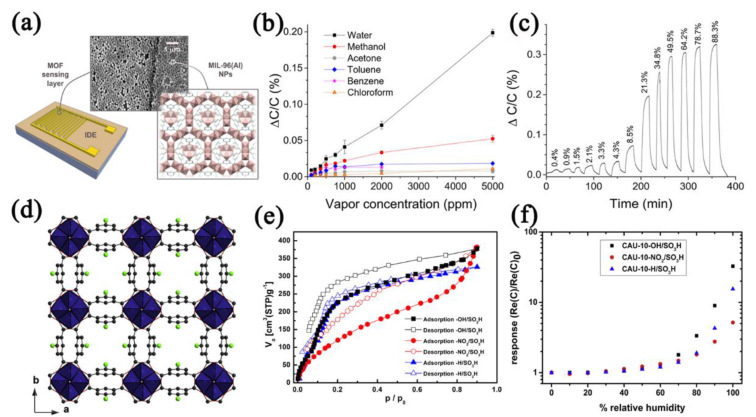
(**a**) MIL−96(Al) structure and SEM image of MIL−96(Al) LB film on the IDE electrode. (**b**) Selectivity of MIL−96(Al)−based sensor to water vapor and other vapors. (**c**) Response curve of MIL−96(Al)−based sensor to humidity in the range of 0.4–88.3% RH. Reproduced with permission from Ref. [54]. Copyright 2020, American Chemical Society. (**d**) Crystal structure of the CAU−10−X with AlO_6_ octahedra helices (blue), isophthalate linkers (black), and position of the functional groups (green). (**e**) Water sorption isotherms of CAU−10−X at 298 K. (**f**) Responses of CAU−10−X−based sensors to humidity in the range of 0–100% RH. Reproduced with permission from Ref. [98]. Copyright 2022, Elsevier.

Al−based MOFs, for example, MIL−96 and CAU−10, are well known for their high stability and strong hydrophilicity, and thus can be used in capacitive−type humidity sensors. Andrés [54] and Rauf [97] prepared MIL−96(Al)−based humidity sensors on IDE electrodes (Figure 10a) and textiles using the Langmuir−Blodgett (LB) method. The MIL−96(Al) IDE sensor showed high selectivity and the LOD reached 0.21% RH (Figure 10b,c). Weiss et al. [98,99] prepared CAU−10−X(Al) with different functional groups (X: −NO_2_, −SO_3_H, −OH, etc.) to research the effect of functional groups on the performance of MOF−based capacitive−type humidity sensors (Figure 10d). At a low water pressure (0–0.05 P/P_0_), MOFs with different groups showed a similar water−adsorbing capacity, and MOFs with high hydrophilic groups (−SO_3_H, −OH) exhibited a stronger water−adsorbing capacity under high water pressure (0.1–0.2 P/P_0_) (Figure 10e). The pore−filling pressure was closely linked to the pore size, and the introduction of the hydrophilic group (−SO_3_H) changed the pore size and water adsorption affinity of CAU−10, leading to stronger adsorbate−adsorbent interactions in low humidity. Thus, the capacitance changes of the CAU−10−X(Al)−based humidity sensors were caused by the changes in dielectric permittivity (Figure 10f).

#### 2.1.4. MOF−Based Resistive−Type Humidity Sensors

One of the most important reasons for the decrease in the resistance of humidity sensors in high humidity is the increase in the conductivity [100]. When the humidity increases, the adsorbed water molecules form a continuous water film on the surface of the sensing materials through hydrogen bonds, which facilitates proton conduction and increases the conductivity of the sensing materials [101]. Through ligand selection and structural modification, MOFs could achieve large conductivity changes under different humidity atmospheres [28,31]. Since the resistance and current can be converted to each other, the MOF−based resistive and current−type humidity sensors are discussed together (Table 3).

**Figure 11:**
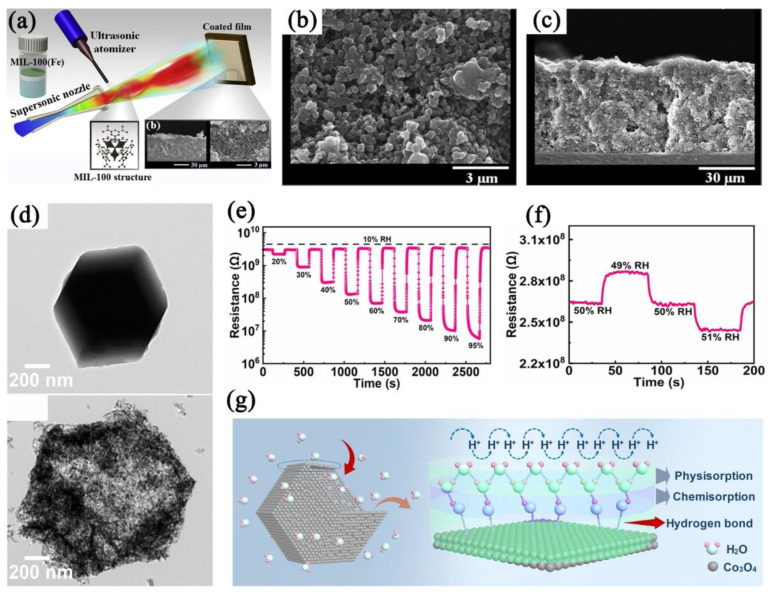
(**a**) Schematic illustration of cold−spray process showing the deposition of MIL−100 film. (**b**) Enlarged surface morphology and (**c**) cross−section of the prepared MIL−100 film. Reproduced with permission from Ref. [105]. Copyright 2017, Elsevier. (**d**) TEM images of ZIF−67− and ZIF−67−derived Co_3_O_4_. (**e**) Response curve of Co_3_O_4_−based humidity sensor to humidity in the range of 10–95% RH. (**f**) Response curve of Co_3_O_4_−based humidity sensor to 1% RH change. (**g**) Schematic diagram of continuous water films and proton conduction on the surface of Co_3_O_4_. Reproduced with permission from Ref. [55]. Copyright 2022, Elsevier.

It is known that 1D channels are conducive to proton conduction [53]. Grag et al. [102,103,104] prepared hydrophilic MOF−76 with three metal centers (Nd, Tb, and Gd). The prepared MOFs possess 1D channels and a small pore size, and were used for humidity sensing. It was found that the water molecules adsorbed by the pores raised the dielectric parameter and decreased the resistance of MOFs, showing the importance of 1D channels in MOFs in humidity sensing. The continuous film also facilitates proton conduction. Lee et al. [105] reported MIL−100 humidity−sensitive film via supersonic spraying (Figure 11a–c). The prepared film was stable in ultrasonic and peel tests, and the prepared sensor exhibited good cycling characteristics (>10 cycles). Smith et al. [106] reported humidity sensors based on the self−assembly of conductive MOFs (Ni_3_HHTP_2_ and Ni_3_HITP_2_) on textiles. The conductivity was 1.6 × 10^−4^ S/cm and 2.6 × 10^−3^ S/cm for Ni_3_HHTP_2_ and Ni_3_HITP_2_−based cotton SOFT sensors, respectively. The sensors showed a response of ~40% to 0–5000 ppm of water molecules, proving the possibility of using MOFs in conductive textiles for wearable humidity sensors. Park et al. [107] used surfactant−induced pre−polymerization at the water surface to form 2D MOF films with a controllable thickness (8–340 nm), and the MOF film can be transferred to different substrates (Si/SiO_2_, Au, etc.) without damage. The 2D MOF−based sensor can detect 1–1000 ppm of water molecules, which the sensor based on MOF powder cannot accomplish.

One of the main ways to reduce the resistance of humidity sensors is to form continuous water film on the surface of materials. Zhang et al. [55] prepared ZIF−67−derived Co_3_O_4_ with mesoporous and hollow structures. The Co_3_O_4_−based sensor is highly sensitive to humidity due to its strong hydrophilicity and 3D porous structure (Figure 11d). At high humidity, water molecules are adsorbed on the surface of Co_3_O_4_ through hydrogen bonds, forming a continuous water film, which is conducive to proton conduction (Figure 11g). The sensor showed the sensing characteristics of small hysteresis (2.6% RH), fast response (1 s), and high resolution (1% RH) in the humidity range of 10–95% RH (Figure 11e,f). Huo et al. [108] prepared a 2D Co−MOF for humidity sensing, but the Co−MOF−based sensor showed no response when the humidity was lower than 90% RH. Co−MOF@PA (PA: phytic acid) was then prepared by post−synthesis. The introduced PA etched the framework and enhanced the hydrophilicity of Co−MOF. Compared to the Co−MOF−based sensor, the Co−MOF@PA−based sensor showed a lower detection limit (23% RH) and higher response (>2000).

**Figure 12:**
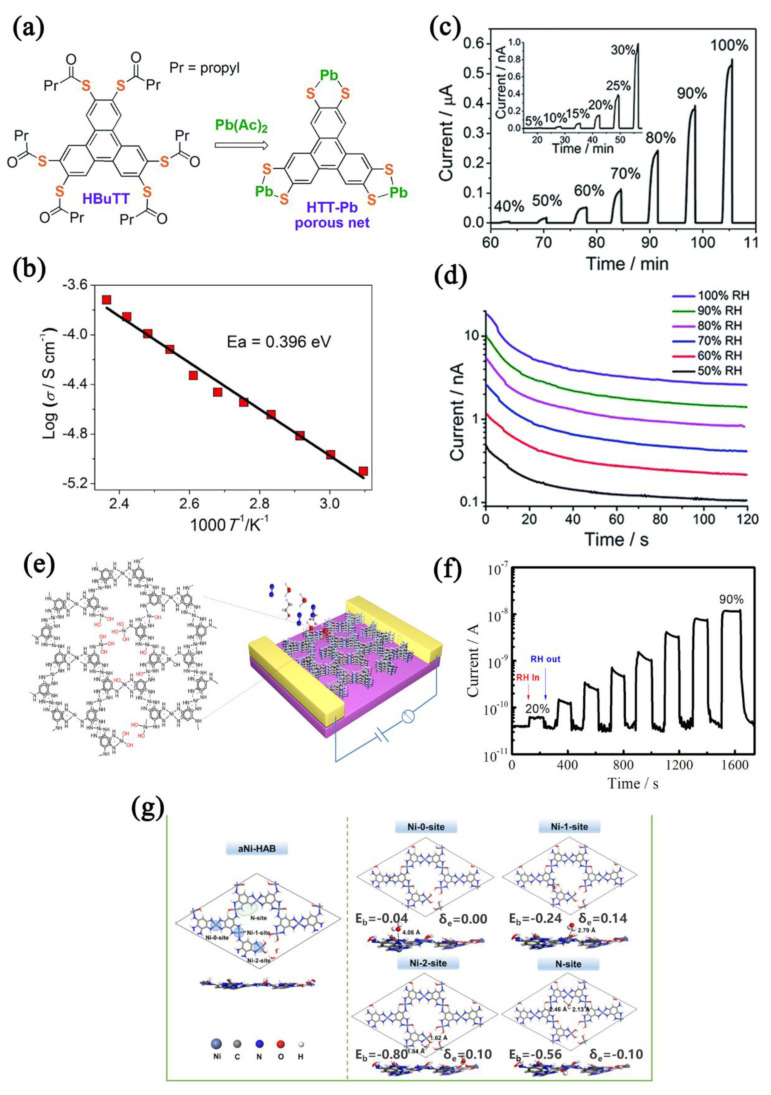
(**a**) Scheme of the synthesis of HTT−Pb framework. (**b**) Calculation of the activation energy of HTT−Pb. (**c**) Response curve of HTT−Pb−based sensor to humidity in the range of 5–100% RH. (**d**) Curves of current vs. time of the HTT−Pb−based sensor at various RH obtained by the DC reverse polarity method. Reproduced with permission from Ref. [109]. Copyright 2017, The Royal Society of Chemistry. (**e**) Crystal structure of the missing−linker amorphous aNi−HAB− and aNi−HAB−based sensor. (**f**) Response curve of aNi−HAB−based sensor to humidity in the range of 0–90% RH. (**g**) Side and top views of a slab model of aNi−HAB with possible H_2_O adsorption sites: purple, gray, blue, red, and white balls correspond to Ni, C, N, O, and H atoms, respectively. Reproduced with permission from Ref. [111]. Copyright 2021, American Chemical Society.

The contribution of different conduction types (ion conduction, electron conduction, proton conduction, etc.) to the change of conductivity can be determined through the instantaneous polarity reversion, which is helpful to further understand the sensing mechanism. Huang et al. [109] synthesized an HTT−Pb (HBuTT: 2,3,6,7,10,11−hexakis(butyrylthio)triphenylene) MOF with a helical topology (Figure 12a). The HTT−Pb film and a humidity sensor with high electrical conductivity (1.1 × 10^−6^ S/cm) at room temperature were obtained (Figure 12b). The conductivity of the humidity sensor increased 10^4^ times when the humidity increased from 5% RH to 90% RH, which was time−dependent (Figure 12c,d), and enabled a fast response (~6 s). Lv et al. [110] reported a 3D MOF (NBu_4_)_2_Cu_2_(dhbq)_3_. The current of the (NBu_4_)_2_Cu_2_(dhbq)_3_−based humidity sensor in dry air reached 10^−12^ A, and the current response showed a four orders of magnitude increase when at 80% RH with good repeatability and a response time of 54 s. Through the instantaneous polarity reversion, it was found that electron conduction and ion conduction both contributed to the change in the conductivity, but mainly electron conduction. Liu et al. [111] prepared a ligand−deficient 2D conductive MOF Ni−HAB using the oxidative synthesis method, and the increased structural defects were beneficial to the adsorption and desorption of water molecules (Figure 12e). The proton conduction−based sensing mechanism endowed the Ni−HAB−based humidity sensor with a high sensitivity (>10^2^) and a fast response (4.9 s) (Figure 12f,g).

### 2.2. MOF−Based Fluorescent−Type Humidity Sensors

Fluorescent MOFs are an important sub−category of MOFs [112,113], and have been widely used in chemical sensing [114,115,116,117]. The rational selection of metal centers and ligands is necessary when constructing fluorescent MOFs for humidity sensing. MOFs with metal centers containing d^10^ orbitals (for example, lanthanide metals (Eu, Tb, Dy) or Zn, Cd, Cu(I), etc.) are potential fluorescent materials [118,119]. In addition, rigid ligands containing aromatic rings are usually used for the synthesis of fluorescent MOFs [120]. Generally speaking, the fluorescence of MOFs comes from ligand−to−ligand charge transfer (LLCT), ligand−to−metal charge transfer (LMCT), or metal−to−ligand charge transfer (MLCT) [121]. In order to achieve humidity detection with fluorescent MOFs, the water molecules should affect the excited states of MOFs efficiently (Table 4).

**Figure 13:**
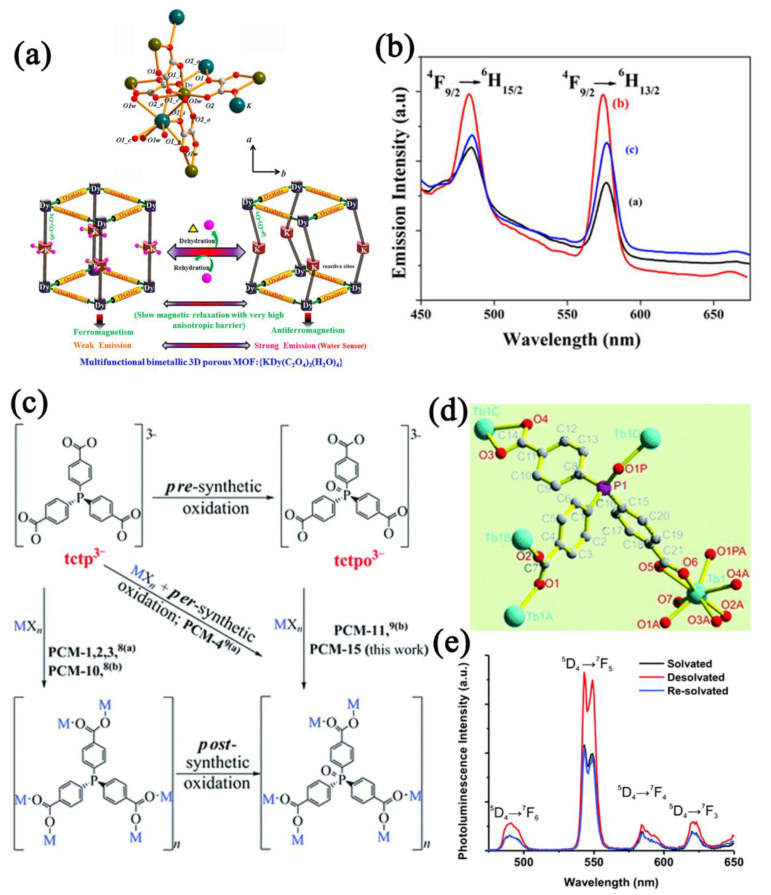
(**a**) Coordination environment and water−induced structural change of Dy−MOF. (**b**) Luminescent spectra (λ_ex_ = 365 nm) of Dy−MOF−based sensor to water (original (**a**, black line), dehydrated (**b**, red line), rehydrated (**c**, blue line)). Reproduced with permission from Ref. [123]. Copyright 2013, American Chemical Society. (**c**) Different methods to introduce the P=O group to prepare PCM−15. (**d**) Coordination environment of PCM−15. (**e**) Photoluminescence spectra for solvated, desolvated, and rehydrated PCM−15−based sensor (λ_ex_ = 330 nm). Reproduced with permission from Ref. [124]. Copyright 2015, The Royal Society of Chemistry.

Utilizing the reversible adsorption and desorption of water molecules by MOFs to affect the excited states of MOFs is an effective way to achieve fluorescent humidity sensing. MOFs with lanthanide metal centers possess unique fluorescence emission peaks, which are easily distinguished. Additionally, water molecules have the ability to influence the luminescence of lanthanide elements because water molecules can affect the energy transfer efficiency between ligands and lanthanide metals. Zhu et al. [122] prepared two 3D fluorescent Ln−MOFs ([Ln_2_(fumarate)_2_(oxalate)(H_2_O)_4_].4H_2_O, Ln = Eu, Tb). The pores of the Ln−MOFs were occupied by water molecules, and the fluorescence was quenched when the water molecules were removed and recovered after hydration. Mohapatra et al. [123] researched the fluorescence changes of Dy−MOF before and after removing the water molecules (Figure 13a,b), and it was considered that the fluorescence changes were related to the changes in the metal coordination environment, which changed the energy transfer from the Dy center to the linker. Ibarra et al. [124] prepared a terbium−doped phosphorus coordination material, PCM−15 (Figure 13c). PCM−15 exhibited terbium−specific luminescence, and since PCM−15 has open metal sites (Figure 13d), the fluorescence intensity greatly increased when the water molecules were removed, showing a specific sensitivity to water molecules (Figure 13e).

The fluorescent MOFs mentioned above showed potential in water molecule detection, but can only be used for the qualitative indication of water molecules. Therefore, more stable and accurate fluorescence humidity sensing is necessary. Yu et al. [125] reported two types of luminescent Ln−MOF, which showed the reversible adsorption and desorption of water molecules with no structure being destroyed. The LOD of Ln−MOF−based sensors reached 5% RH and realized visual inspection. Wang et al. [126] reported a luminescent Eu−MOF filled with water molecules within 1D channels. The effect of the water molecules on the fluorescence intensity of the Eu−MOF was determined by the combination of thermogravimetry and fluorescence. When water molecules escaped from the channel, the thermal vibrations of the framework were stronger, reducing the energy transfer efficiency from the ligands to the Eu^3+^ and resulting in decreased fluorescence intensity. The fluorescence intensity of the Eu−MOF showed a linear increase from 33% RH to 85% RH. Considering that some pure MOFs are unstable in a humid environment, Stangl et al. [127] encapsulated Ln−MOFs into the mixed matrix membrane. Compared to the bulk MOFs, the composites were more stable in the humid environment and showed the reversible detection of humidity within 24 h (12–50% RH).

**Figure 14:**
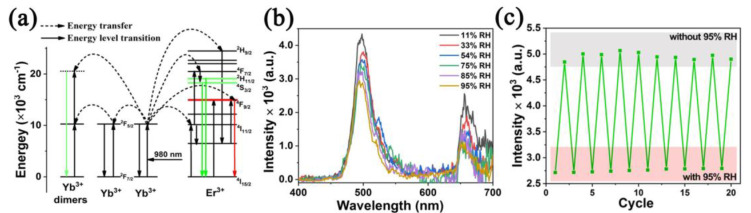
(**a**) Schematic energy−level diagram of Y/Yb/Er−MOF with the possible energy transfer paths. (**b**) Luminescent spectra (λ_ex_ = 980 nm, 1.5 W) of Y/Yb/Er−MOF−based sensor to humidity in the range of 11–95% RH. (**c**) Cycling stability of Y/Yb/Er−MOF−based sensor. Reproduced with permission from Ref. [129]. Copyright 1996–2022, MDPI, Basel, Switzerland.

Introducing functional groups or tuning the metal coordination environment can improve the cycling performance of Ln−MOFs. Gao et al. [128] prepared a highly stable Eu−MOF ([Eu(H_2_O)_2_(mpca)_2_Eu(H_2_O)_6_M(CN)_8_]·nH_2_O) containing cyano groups. The introduced cyano group and carboxylic acid group can form hydrogen bonds with water molecules. The linear detection range of the Eu−MOF−based sensor was 0–100% RH with excellent recycle ability (at least 7000 s). Wang et al. [129] prepared an upconversion luminescent Ln−MOF (Y/Yb/Er−MOF). The water molecules absorb the energy transferred from Yb, thus quenching the upconversion luminescence of the Ln−MOF (Figure 14a). The fluorescence intensity of the sensor was quenched linearly from 11% RH to 95% RH and showed excellent cycling characteristics (at least 20 cycles) (Figure 14b,c).

**Figure 15:**
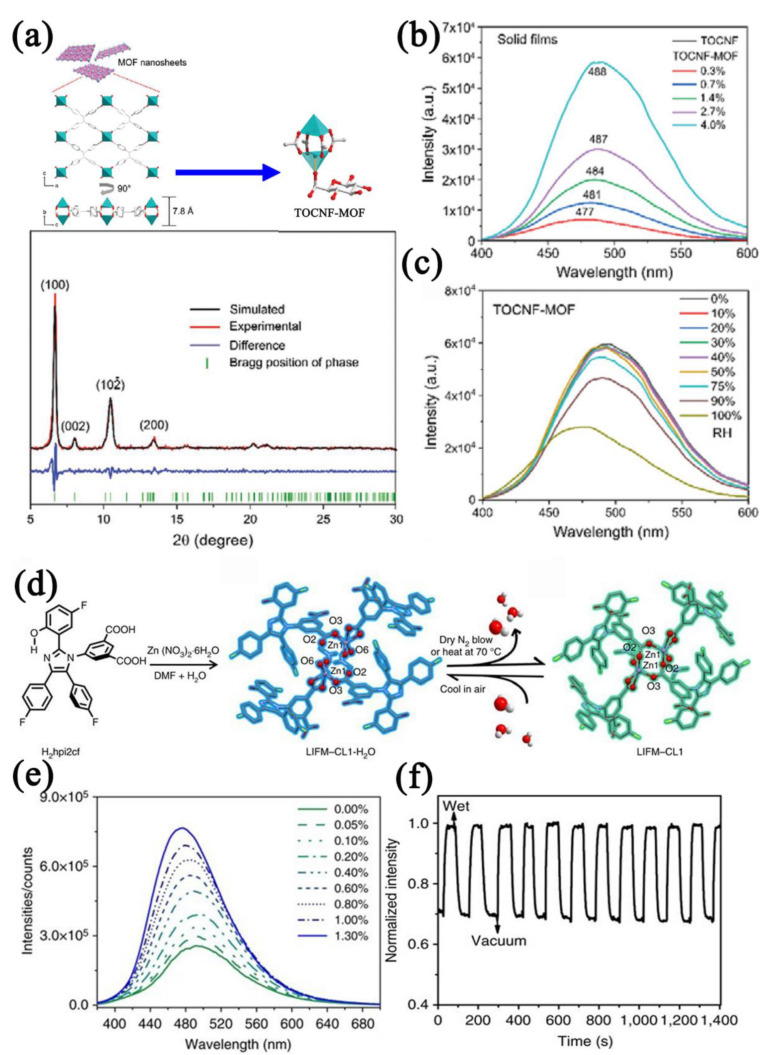
(**a**) Schematic diagram of ultrathin MOF nanosheets and TOCNF−MOF and the XRD patterns of MOF nanosheets. (**b**) Luminescent spectra (λ_ex_ = 365 nm) of TOCNF−MOF−based sensor in different MOF contents (0.3–4.0 wt%). (**c**) Luminescent spectra (λ_ex_ = 365 nm) of TOCNF−MOF−based sensor in the humidity ranges of 0–100% RH. Reproduced with permission from Ref. [56]. Copyright 2022, Wiley−VCH GmbH. (**d**) Synthetic route and structural transformation of Zn−MOF (left: ligand, middle: hydrated LIFM−CL1−H_2_O (blue), right: dehydrated LIFM−CL1 (cyan)). (**e**) Luminescent spectra (λ_ex_ = 365 nm) of Zn−MOF−based sensor to different water contents (0–1.3% *v*/*v*). (**f**) Cycling curve of Zn−MOF−based sensor between wet or vacuum atmospheres. Reproduced with permission from Ref. [131]. Copyright 2017, Springer Nature.

MOFs that combine O−donor or N−donor ligands and transition metals that own d^10^ configurations (Zn, Cu, etc.) exhibit appealing structures as well as photoluminescence properties. Wang et al. [130] reported a luminescent MOF [Zn(dpe)(bdc)]·4H_2_O (dpe = 1,2−bis(4−pyridyl)ethane, bdc^2−^ = dianion of benzenedicarboxylic acid) with a unique 2D water layer. The emission peak and intensity of the MOF changed with the humidity. Tan et al. [56] reported aggregation−induced−emission luminogen (AIEgen) MOF nanosheets and TOCNF−MOF film−based humidity sensors (Figure 15a). Since the water molecules inhibited the motion of the TPE rotor in AIEgen, more energy was released in the form of fluorescent radiation. The fluorescence intensity of AIEgen MOF nanosheets was enhanced with the increased humidity. Cellulose nanofibril (CNF) is hygroscopic and swells after absorbing water molecules; the swollen CNF could change the nanosheet spacing and reduce the fluorescence intensity. The TOCNF−MOF−based humidity sensor showed fluorescent quench in the humidity range of 0–100% RH (Figure 15b,c). Chen et al. [131] reported a robust microporous MOF Zn(hpi2cf)(DMF)(H_2_O) (H_2_hpi2cf=5−(2− (5−fluoro−2−hydroxyphenyl)−4,5−bis(4−fluorophenyl)−1H−imidazol−1−yl)isophthalic acid). MOFs undergo single crystal−to−single crystal (SC−SC) transition upon dehydration because of the change in the fine arrangement of Zn−O clusters, and the fluorescence properties are changed (Figure 15d). The MOF−based fluorescent sensor exhibited an intensity increase when the water concentration increased, and the LOD was as low as 1% RH with good cycle characteristics (Figure 15e,f). Fluorescent MOFs with main metals also have the potential for water detection. Douvali et al. [132] reported a 3D open−skeleton MOF (Mg(H_2_dhtp)(H_2_O)_2_)⋅DMAc with structure breathing ability. The host−guest interaction between the water molecules and the MOF framework activates the fluorescence intensity, realizing the detection of extremely low water concentrations in organic solvents. The detection range was calculated to be 0.05–5% *v*/*v*.

**Figure 16:**
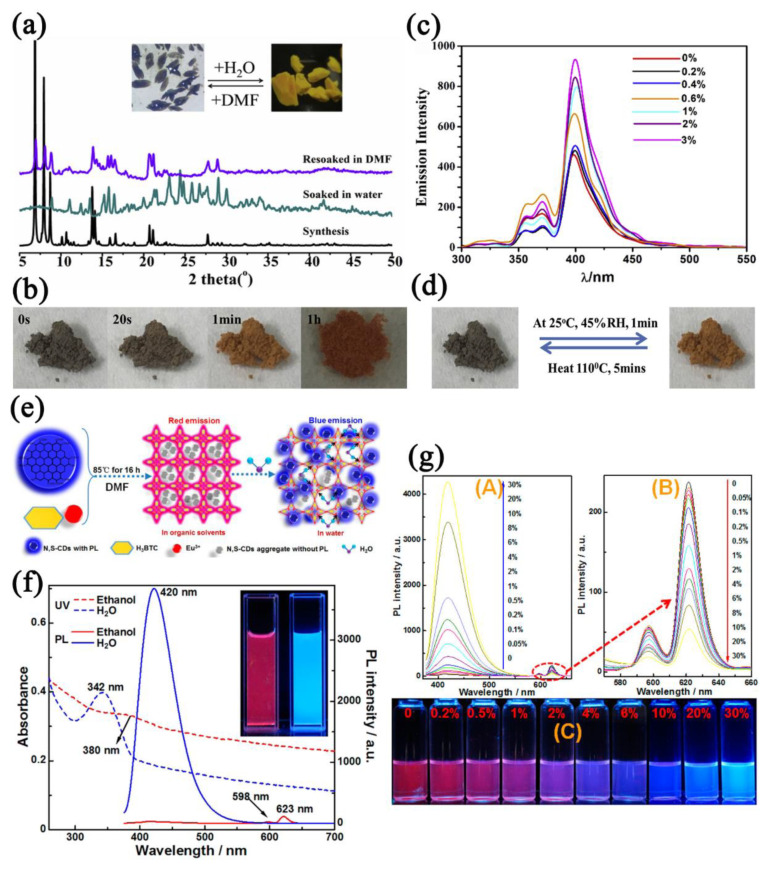
(**a**) XRD patterns and photographs of Co−MOF soaked in DMF and water. (**b**) Photograph images of Co−MOF at 45% RH for different times. (**c**) Luminescent spectrum (λ_ex_ = 285 nm) of Co−MOF to different water concentrations (0–3% *v*/*v*) in DMF. (**d**) Photograph images of reversible color changes of Co−MOF at 45% RH. Reproduced with permission from Ref. [134]. Copyright 2019, Elsevier. (**e**) Schematic of the synthesis of Eu−MOF/N,S−CDs and detection of water in organic solvent (**f**) PL emission spectra, UV−Vis absorption spectra, and photographs of Eu−MOF/N,S−CDs in ethanol and water. (**g**) A for the luminescent spectra (λ_ex_ = 365 nm) of Eu−MOFs/N,S−CDs under different water concentrations (0–30% *v*/*v*) in ethanol, B for the amplification of the red emission peaks in (A), C for the photographs (under 365 nm) of Eu−MOFs/N,S−CDs dispersed in ethanol with various water contents (*v*/*v*). Reproduced with permission from Ref. [139]. Copyright 2016, American Chemical Society.

The ratiometric detection of water by fluorescent MOFs is also attractive. It is known that water molecules affect the charge transfer process (LLCT, LMCT, MLCT) of MOFs during photoluminescence. The introduction of color−changing functional groups, encapsulation of guests, and introduction of multiple emission centers in MOFs may be beneficial to the ratiometric detection of water. Drache et al. [133] reported a Zr−MOF DUT−122 introduced with solution−chromic functional groups. The hydrogen bonds between the water molecules and the functional groups induced vibrational coupling, leading to non−radiative transitions that affect the luminescence of MOF. The DUT−122−based sensor exhibited increased fluorescence intensity in the humidity range of 0–100% RH. Qin et al. [134] prepared a naphthalenediimide−based Co−MOF {[Co_2_(DPNDI)(2,6−NDC)_2_]·7(DMF)}*_n_*. The reaction between the DPNDI group and water molecules affected the electronic transfer in the MOF, resulting in solvatochromism (Figure 16a). The Co−MOF−based sensor showed increased fluorescence intensity in the water concentration range of 0–3% *v*/*v* and was visible to the naked eye. In addition, the fluorescence intensity and crystal color of the sensor were time−dependent (Figure 16b–d).

The difference between the encapsulated guests and the electron transfer between the guests and MOFs also affect the emission color of the MOFs in humidity. Yu et al. [135] reported a Cu(I) MOF (CH_3_CN·MeOH·1.5H_2_O⊂Cu_2_(L)_2_I_2_). Since the structural parameters of the MOF changed under the action of humidity, different guests (H_2_O or DMF) were encapsulated. The MOF−based sensor showed different degrees of color changes under humid atmospheres (33–57% RH) and the color was time−dependent. Yin et al. [136] prepared MOF MIL−101(Al)−NH_2_ with Ru(bpy)_3_^2+^ encapsulated in the pores. Under different humidity levels, the Al centers and Ru(bpy)_3_^2+^ were considered to transfer charges to ligands, respectively, realizing the color transition of MOF. The Ru@MIL−101(Al)−NH_2_−based sensor achieved wide−ranging (0–100% *v*/*v*) and fast−response (<1 min) detection of water molecules in solvents.

When partial emission is suppressed by water molecules, fluorescent MOFs with multiple emission centers will exhibit another color of emission, resulting in a colorimetric response to humidity. Wehner et al. [137] prepared a fluorescence humidity sensor with Fe_3_O_4_/SiO_2_ as the core and Ln−MOF as the shell. Utilizing the oxophilicity of lanthanide metals and different luminescent centers, the MOF−based fluorescence sensor realized ratiometric and colorimetric water detection, and the LOD reached 0.03 wt% (20 μg). Wu et al. [138] prepared a Tb^3+^ and carbon dots (CDs)−doped MOF, Tb^3+^@p−CDs/MOF, in which Tb^3+^ emitted green light and CDs emitted red light. When water molecules were adsorbed, the CDs aggregated and the red light was quenched while the green light was maintained. The ratio of the light intensity at 545 nm to that at 605 nm of the Tb^3+^@p−CDs/MOF−based sensor increased linearly with the humidity, which increased from 33.0% RH to 85.1% RH. Dong et al. [139] prepared a nitrogen and sulfur co−doped carbon dots (N,S−CDs)−encapsulated europium MOF (Eu−MOF/N,S−CD). After adsorbing water molecules, the red light of the Eu−MOF was quenched by O−H species, while the blue light emitted by the CDs was not affected (Figure 16e). The Eu−MOF/N,S−CD−based sensor realized the colorimetric detection of water molecules in ethanol (Figure 16f); the ratio of the luminescence intensity at 420 nm to that at 623 nm increased linearly with the increasing water content in ethanol in the range of 0.05–4% *v*/*v*, and the LOD was calculated to be 0.03% *v*/*v* (Figure 16g).

### 2.3. MOF−Based QCM−Type Humidity Sensors

**Figure 17:**
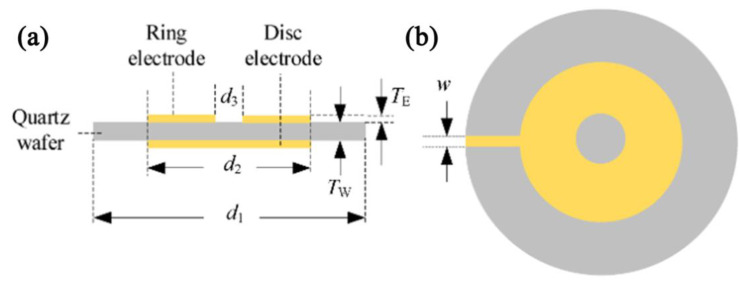
Structure of QCM element: side view (**a**), top view (**b**). Reproduced with permission from Ref. [140]. Copyright 1996–2022, MDPI, Basel, Switzerland.

A QCM is a mass−sensitive device capable of measuring tiny mass changes on a nanogram scale (Figure 17) [140]. It owns the advantages of a low detection limit, wide detection range, and high accuracy. Normally, a single QCM element has no function, so a sensing layer needs to be loaded on the QCM element [141].

The working principle of the QCM−type humidity sensor is the change of the resonance frequency caused by the mass change of the sensing material in different humidity atmospheres [142], so the sensing performance depends on the water adsorption capacity of the sensing materials. The change in mass (Δm) on the surface of the QCM is related to the shift in resonance frequency (Δf) given by the Sauerbrey equation (Equation (3)) [141].

(3) $$\Delta f=-\left(\frac{2f_{0}^{2}}{A\sqrt{\rho_{q}\mu_{q}}}\right)\Delta m$$

where $f_{0}$ is the basic frequency of the QCM element, A is the electrode area, $\rho_{q}$ is the density of quartz crystals (2.649 g/cm^−3^), and $\mu_{q}$ is the shear modulus (2.947 × 10^10^ N/m^2^). The sensitivity of QCM−type humidity sensors is defined as the change in frequency of the QCM element for every 1% RH change. MOFs have the characteristics of a large specific surface area and many active sites. Thus, MOF−based QCM sensors can also be utilized for humidity detection (Table 5).

**Figure 18:**
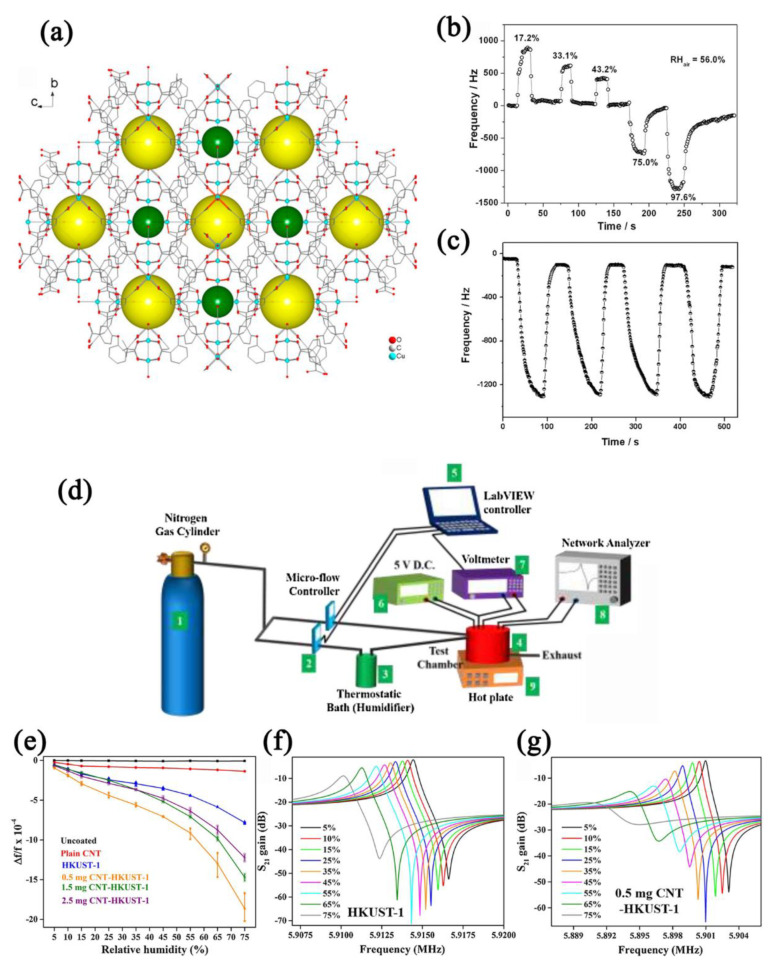
(**a**) Crystal structure of [Cu_3_L_2_(H_2_O)_2_._75_]·0.75H_2_O·1.75DMA. (**b**) Response curve of Cu−MOF−based QCM sensor to humidity in the range of 17.2–97.6% RH. (**c**) Cycling curve of Cu−MOF−based QCM sensor. Reproduced with permission from Ref. [57]. Copyright 2017, American Chemical Society. (**d**) Schematic of the experimental setup for characterizing the QCM−coated humidity sensor. (**e**) Frequency response curves of CNT−HKUST−1−based QCM sensors to humidity in the range of 5–75% RH. Response curves of (**f**) HKUST−1−based QCM sensor and (**g**) 0.5 mg CNT−HKUST−1−based QCM sensors to humidity in the range of 5–75% RH. Reproduced with permission from Ref. [144]. Copyright 2018, Elsevier.

Copper−based MOFs attract wide attention for QCM−type humidity sensors. Zhou et al. [57] reported a QCM humidity sensor based on a copper MOF [Cu_3_L_2_(H_2_O)_2_._75_]·0.75H_2_O·1.75DMA (Figure 18a). The sensor showed a 4162 Hz of frequency shift to humidity change from 17.2% RH to 97.6% RH with a sensitivity of 28.7 Hz/% RH (Figure 18b), and the corresponding response time and recovery time were calculated to be 30 s and 18 s, respectively (Figure 18c). Kosuru et al. [143] fabricated QCM humidity sensors by dripping PVP and HKUST−1 onto QCM elements, respectively. The QCM element, PVP QCM sensor, and HKUST−1 QCM sensor showed different responses (7 Hz, 48 Hz, and 720 Hz, respectively) in the humidity range of 22–68% RH. Through theoretical calculation, it was found that the porosity, hydrophilicity, and large adsorption energy endowed the HKUST−1 QCM with sensor better sensing properties. Chappanda et al. [144] researched the humidity−sensing performance of CNT−HKUST−1−based QCM sensors (CNT: carbon nanotube, Figure 18d). The CNT affected the nucleation of HKUST−1, resulting in a larger surface area of HKUST−1. The sensitivity of the CNT−HKUST−1 QCM sensor reached 141 Hz/% RH in the humidity range of 5–75% RH (Figure 18e–g), with a hysteresis of 5% RH and excellent long−term stability (>10 days).

MOF derivatives were also utilized in QCM−type humidity sensors. Zhang et al. [145] prepared MOF−derived humidity−sensing materials (ZnCo_2_O_4_/polypyrrole (PPy) composites) by in situ polymerization. The porous nanostructure of the ZnCo_2_O_4_ and the swelling effect of PPy provide more water adsorption sites. The sensitivity of the QCM sensor was 58.4 Hz/% RH in the humidity range of 0–95% RH, with a small hysteresis (3.9% RH) and a fast response (8 s). Zhang et al. [146] prepared MOF−derived TiO_2_ QCM humidity sensors with three morphologies. Among them, the sensor with a hollow spherical morphology (the highest specific surface area) possessed the best sensing characteristics including high sensitivity (33.8 Hz/% RH), fast response (5 s), and good stability (>30 days). What is more, Chen et al. [147] prepared a QCM humidity sensor with a MOF−derived SnO_2_/chitosan (CS) nanostructure. The particle size of the SnO_2_ NPs was ~20 nm, which is beneficial to generate a large specific surface area. Therefore, the MOF−SnO_2_/CS−based QCM sensor exhibited a high sensitivity (~43.14 Hz/% RH) to humidity change (0–97% RH).

### 2.4. Other MOF−Based Types of Humidity Sensors

MOF−based electrically transduced humidity sensors, fluorescent−type humidity sensors, and QCM−type humidity sensors are able to detect humidity in most application scenarios. In addition, the MOF−based humidity sensors used in some special conditions still need to be researched (for example, environments with flammable and explosive substances). There are several types of MOF−based humidity sensors that can be used in special scenarios (Table 6).

**Figure 19:**
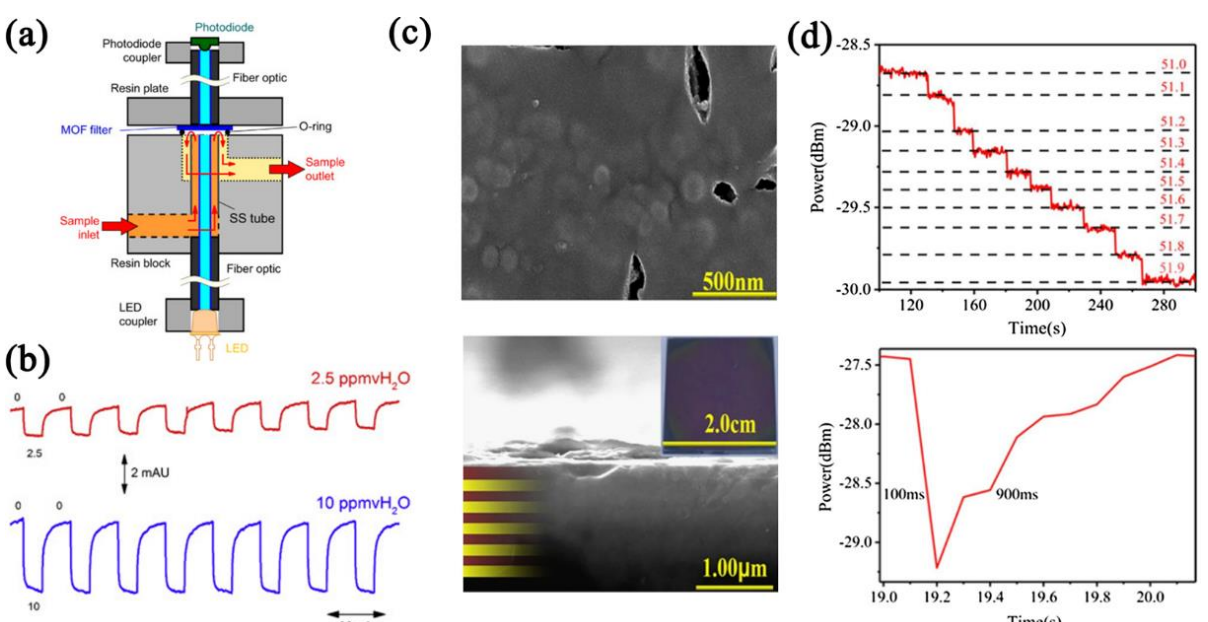
(**a**) Schematic of the fiber−optic humidity−sensing device. (**b**) Cycling curves of Cu−BTC−based fiber−optic humidity sensor in low ppmv levels (0–2.5 ppmv and 0–10 ppmv). Reproduced with permission from Ref. [148]. Copyright 2015, Elsevier. (**c**) Above sectional and cross−sectional SEM images and optical image of MOF−801/TiO_2_ 1DPC. (**d**) Response curve of MOF−801/TiO_2_ 1DPC sensor in 0.1% RH change and the response time and recovery time of MOF−801/TiO_2_ 1DPC sensor. Reproduced with permission from Ref. [150]. Copyright 2020, Elsevier.

Optical fiber has the advantages of anti−electromagnetic interference and corrosion resistance, and has been widely used in the detection field [154,155,156]. Normally, fiber−based humidity sensors mainly include Mach−Zehnder interferometers (MZI), Fabry−Perot interferometers (FPI), photonic crystal fibers (PCF), and long−period fiber gratings (LPFG) [157]. The working principle is the change in volume and refractive index of the sensing materials after adsorbing water molecules. Ohira et al. [148] prepared a Cu−BTC−based fiber−optic humidity sensor with Fabry−Perot interferometers (FPI) (Figure 19a,b). The prepared sensor showed the ability to detect water concentrations as low as 40 ppb. In addition, after adsorbing the water molecules, the coordination reaction between the copper ions and water molecules changed the color of the Cu−BTC, which can achieve visual detection. Yan et al. [149] fabricated a GO/Co−MOF−74−based long−period fiber grating (LPFG) sensor using the coating method. The refractive index and the electrical conductivity of the GO decreased after adsorbing water molecules, but the refractive index of the Co−MOF−74 increased. Based on these, the GO/Co−MOF−74−based sensor exhibited a two−stage signal change.

Photonic crystal humidity sensors are also attractive among fiber−optic humidity sensors. Zhan et al. [150] designed a photonic crystal humidity sensor with strong hydrophilicity using MOF−801 and TiO_2_ (Figure 19c). MOF−801 adsorbs water molecules and TiO_2_ provides a high refractive index contrast, resulting in a redshift of the maximum reflection peak. The sensor showed a linear response (R^2^ = 0.989) in the humidity range of 20–90% RH, with a resolution of 0.1% RH, a sensitivity of 0.119 nm/% RH, and an ultrafast response speed (0.1 s) (Figure 19d). Chen et al. [151] prepared a silica colloidal photonic crystal self−assembly and Cu−BTC was compounded onto the crystals to improve the hydrophilicity, and the relay adsorption of water molecules between the Cu−BTC and silica colloidal photonic crystals expanded the humidity−sensing range (500–20,000 ppm).

Microwave sensors are highly compatible with other circuit modules and are sensitive to environmental changes or the electromagnetic property changes of surrounding materials [158], making them a potential category of humidity sensors. Yu et al. [58] prepared a microwave humidity sensor with carbon dots−modified MOF−derived porous Co_3_O_4_. The changes in the electrical conductivity and dielectric properties of the sensor after adsorbing water can be detected by the microwave resonator. The sensor showed a sensitivity of 3.40 MHz/% RH in a wide humidity change (5–99% RH) with a fast response (<5 s).

Surface acoustic wave (SAW) sensors are also used in humidity sensing [159]. When the sensing materials adsorb water molecules, the mass and conductivity of the sensing layer change accordingly, shifting the frequency of the surface acoustic wave. Robinson et al. [152] fabricated Cu−BTC SAW devices by the layer−by−layer method and the sensing films with 20–100 cycles exhibited the best sensing properties. The detection range of the sensor was 3–14,800 ppm, and the adsorption order of Cu−BTC was determined (copper sites first, pores second, and finally, fully saturated).

For a qualitative indication of water molecules, Ullman et al. [153] fabricated humidity sensors on different substrates by spin−coating HKUST−1. The reflectance spectrum of HKUST−1 changed after adsorbing water molecules. Since the reflection intensity of HKUST−1 was highly sensitive to humidity changes, the HKUST−1−based sensor showed a good response (>100% at 525 nm) in the humidity range of 1–5% RH.

### 2.5. Comparison of Different MOF−Based Humidity Sensors

As summarized above, with the development of material science, the humidity−sensing performance indexes of MOF−based humidity sensors have been greatly improved. Different types of humidity sensors own different technical characteristics, while different applications have different requirements. In order to find the most suitable type of humidity sensor to meet the actual requirements, it is reasonable to compare the sensing performance indexes of the different types of MOF−based humidity sensors (Table 7).

Among all types of MOF−based humidity sensors, impedance−type humidity sensors possess smaller hysteresis, better linearity, and a shorter response time, but the structure of this type of sensor restricts their application in detecting water molecules in liquid media. Capacitive−type humidity sensors can detect low concentrations of water molecules, but with worse linearity, and resistive−type humidity sensors have the characteristic that can work under direct current with a fast recovery time. Fluorescent−type humidity sensors are widely used in the detection of water molecules in solvents, but the poor circulation, linearity, and long response time limit their wider application. QCM−type humidity sensors can be used for low humidity detection, although their poor selectivity, the difficulties in miniaturizing and integrating them, and their high cost limit their application scenarios. Fiber−optic−type humidity sensors can achieve high resolution humidity detection with high stability, but the special materials, high cost, and low sensitivity limit their application.

## 3. Summary and Outlook

In summary, the research on humidity sensors based on MOFs has made promising progress in recent years. The impedance−type, capacitive−type, resistive−type, fluorescence−type, QCM−type, and other types of humidity sensors based on MOFs have been successfully prepared. There are some common and unique features among the different types of MOFs−based humidity sensors.

Similarities:

(1) Strong hydrophilicity. When the detection takes place in a low humidity range, strong hydrophilicity can help the sensor to operate effectively, and when the detection is in full humidity range, strong hydrophilicity can make the sensor more sensitive.
(2) High porosity. The adsorption and diffusion of water molecules and the transmission of electron signals all require channels, and the selectivity of sensors can also be improved by the design of the channels.
(3) Adjustable structure. One of the most attractive features of MOFs among humidity−sensing materials is the adjustable structure, which allows researchers to prepare MOFs for different requirements (porosity, hydrophilicity, etc.).
(4) Good stability. For practical and user−friendly humidity sensors, reliability and recyclability are necessary. The structural stability of MOFs is the premise of stable sensors.

Differences:

(1) Connection. For electrically transduced humidity sensors, a good connection is needed. The adsorption, diffusion, signal conversion, conduction, and device fabrication of water molecules all put forward a higher connection in sensing films.
(2) Conductivity. For electrically transduced humidity sensors and SAW sensors, the changes in conductivity caused by the adsorbed water molecules can be converted into readable signals.
(3) Luminescence. For fluorescent MOF−based humidity sensors, the luminescent property was influenced by metal centers and ligands. Specifically, metal centers influence the intensities, the position of emission peaks, and the emission colors, and ligands also show influence in charge transfer or host−guest interaction.
(4) Optical properties. For MOF−based fiber−optic humidity sensors, the change of refractive index is very important, which requires the optical performance of MOFs.

In addition, there are still many worthwhile directions to be pursued for researchers in material design and device development.

(1) How to combine characterization, data, and simulation calculations to reveal the in−depth sensing mechanism of MOF−based humidity sensors and understand the structure−performance relationship.
(2) How to screen existing MOFs or predict potential MOFs as sensitive materials for humidity sensors based on their structural or fundamental properties.
(3) How to develop humidity−sensitive MOFs with better performance in extreme environments (extremely low concentrations, low/high temperature, etc.).
(4) How to further improve the selectivity of MOF−based humidity sensors.

These challenges are ubiquitous and need to be addressed urgently in MOF−based humidity sensors. The in−depth research of these issues will provide new understanding and strategies for the development of MOF−based humidity sensors.

## Figures and Tables

**Table 1:** Sensing properties of MOF−based impedance−type humidity sensors.

Materials	Humidity Range	Response/ Sensitivity	Frequency	Linearity (R^2^)	Hysteresis	Response Time	Ref.
Fe−BTC	0–2.5 vol% H_2_O	30.4 MΩ/ vol% H_2_O	1 Hz	None	None	None	[76]
NH_2_−MIL−125(Ti)	11–95% RH	27.5	100 Hz	None	<5% RH	45 s	[77]
[Cd(TMA)(DPP)_0.5_∙ H_2_O]*_n_*	11–97% RH	352	100 Hz	None	<2% RH	11 s	[78]
MIL−101(Cr)	33–95% RH	1932	100 Hz	None	4% RH	17 s	[79]
HKUST−1 nanosheet	11–95% RH	>100	None	None	None	2 s	[80]
FeCl_3_−NH_2_− MIL−125(Ti)	11–95% RH	367	100 Hz	None	None	11 s	[81]
LiCl@UiO−66−NH_2_	11–95% RH	10^4^	100 Hz	0.994	4% RH	6 s	[82]
KOH/M050	20–90% RH	0.056 log Z/% RH	1 kHz	0.930	<2% RH	36 s	[83]
U−DPA−Zn	11–97% RH	8070	100 Hz	0.994	1.8% RH	2 s	[84]
IL_15_−MOF−801	0–35% RH	20.3	1 kHz	None	1% RH	0.4 s	[53]
UiO−66−SO_3_Na	33–95% RH	165	1 kHz	0.994	1.2% RH	3.1 s	[85]
IL−UiO−66−NH_2_	5–30% RH	220%	1 kHz	0.994	0.7% RH	2.7 s	[86]
SPEEK/MNS−30%	11–95% RH	2.8 × 10^5^	100 Hz	0.978	3% RH	9 s	[87]
PB−derived Fe_2_O_3_	11–95% RH	1568	50 kHz	0.992	0.59% RH	<2 s	[88]

**Table 2:** Sensing properties of MOF−based capacitive−type humidity sensors.

Materials	Humidity Range	Response/ Sensitivity	Frequency	Linearity (R^2^)	Hysteresis	Response Time	Ref.
Fe−BTC	0–2.3 vol% H_2_O	4.4 pF	1 Hz	None	None	None	[76]
NH_2_−MIL−125(Ti)	11–75% RH	5397%	20 Hz	0.995	1.9% RH	75 s	[92]
[K_8_(ptca)_3_(H_3_O)_4_]*_n_*	20–85% RH	10^5^	100 Hz	None	None	None	[93]
Cu_3_(BTC)_2_	11.3–84.3% RH	123 pF	1000 kHz	0.992	None	20 s	[94]
Cu(bdc)·xH_2_O	11–100% RH	~0.22 pF	1 MHz	0.979	None	None	[95]
Cu−BTC	20–100 ppm	1.13 pF/ppm	1 MHz	None	None	mins	[96]
MIL−96(Al)	0–5000 ppm	0.0088%/ppm	100 kHz	0.991	None	None	[54]
MIL−96(Al)	0.7–90% RH	0.6 fF/% RH	100 kHz	None	None	None	[97]
CAU−10−X(Al)	10–70% RH	~100	100 kHz	None	None	None	[98]
CAU−10−X(Al)	0–100% RH	~10	1039 Hz	None	None	None	[99]

**Table 3:** Sensing properties of MOF−based resistive−type humidity sensors.

Materials	Humidity Range	Response/ Sensitivity	Linearity (R^2^)	Hysteresis	Response Time	Ref.
MOF−76(Tb)	11–98% RH	1.6	0.906	None	None	[102]
MOF−76(Nd)	11–98% RH	20.2	None	None	None	[103]
MOF−76(Gd)	11–98% RH	None	None	None	11 s	[104]
MIL−100(Fe)	60–80% RH	20%	None	None	20 s	[105]
Ni_3_HHTP_2_	0–5000 ppm	40%	None	None	None	[106]
HIB−Cu	1–1000 ppm	8	None	None	21 s	[107]
MOF−derived Co_3_O_4_	10–95% RH	2730	0.993	2.6% RH	1.0 s	[55]
Co−MOF@PA	23–95% RH	2250	None	None	179 s	[108]
HTT−Pb	5–100% RH	10^4^	None	None	6 s	[109]
(NBu_4_)_2_Cu_2_(dhbq)_3_	30–90% RH	2.1 × 10^4^	0.989	None	54 s	[110]
Ni−HAB	20–90% RH	>100	None	None	4.9 s	[111]

**Table 4:** Sensing properties of MOF−based fluorescent−type humidity sensors.

Materials	Metal Center	Humidity Range	Linearity (R^2^)	Fluorescence Change	Ref.
[Ln_2_(fumarate)_2_(oxalate) (H_2_O)_4_].4H_2_O	Eu Tb	None None	None	Quenched Quenched	[122]
{KDy(C_2_O_4_)_2_(H_2_O)_4_}*_n_*	Dy	None	None	Increased	[123]
PCM−15	Tb	None	None	Increased	[124]
Ln−MOF	Eu Tb	5–85% RH 5–85% RH	None	Increased Increased	[125]
{[Eu_2_(L)_3_·(H_2_O)_2_·(DMF)_2_]· 16H_2_O}*_n_*	Eu	33–85% RH	0.950	Increased	[126]
[Ba_0.98_Eu_0.02_(Im)_2_]@PSF [Sr_0.90_Eu_0.10_(Im)_2_]@PSF	Eu Eu	12–50% RH 12–50% RH	None	Quenched Quenched	[127]
[Eu(H_2_O)_2_(mpca)_2_Eu(H_2_O)_6_M(CN)_8_]· nH_2_O	Eu	0–100% RH	None	Quenched	[128]
Y/Yb/Er−MOF	Y, Yb, Er	11–95% RH	0.996	Quenched	[129]
[Zn(dpe)(bdc)]⋅4H_2_O	Zn	None	None	Quenched	[130]
TOCNF−MOF	Zn	0–100% RH	None	Quenched	[56]
Zn(hpi2cf)(DMF)(H_2_O)	Zn	0–1.3% *v*/*v*	0.935	Increased	[131]
[Mg(H_2_dhtp)(H_2_O)_2_]·DMAc	Mg	0–5% *v*/*v*	None	Increased	[132]
DUT−122	Zr	0–100% RH	None	Increased	[133]
[Co_2_(DPNDI)(2,6−NDC)_2_]⋅ 7(DMF)}*_n_*	Co	0–3% *v*/*v*	None	Increased	[134]
CH_3_CN·MeOH·1.5H_2_O⊂ Cu_2_(L)_2_I_2_	Cu(I)	33–78% RH	None	Quenched	[135]
Ru@MIL−101(Al)−NH_2_	Al	0–100% *v*/*v*	0.991	Increased	[136]
Eu−bipy,Tb−bipy@ Fe_3_O_4_/SiO_2_ Eu−BDC,Tb−bipy@ Fe_3_O_4_/SiO_2_	Eu Tb	0–10 wt% 0–10 wt%	None	Quenched Quenched	[137]
Tb^3+^@p−CDs/MOF	Tb/CDs	33–85% RH	0.96	Quenched	[138]
Eu−MOF/N,S−CD	Eu/CDs	0.2–30% *v*/*v*	None	Quenched	[139]

**Table 5:** Sensing properties of MOF−based QCM−type humidity sensors.

Materials	Humidity Range	Response/ Sensitivity	Linearity (R^2^)	Hysteresis	Response Time	Ref
[Cu_3_L_2_(H_2_O)_2.75_]· 0.75H_2_O·1.75DMA	17.2–97.6% RH	28.7 Hz/% RH	0.993	None	30 s	[57]
HKUST−1	22–68% RH	720 Hz	0.990	None	1676 s	[143]
CNT−HKUST−1	5–75% RH	141 Hz/% RH	None	<5% RH	4.2 min	[144]
MOF−ZnCo_2_O_4_/PPy	0–97% RH	58.4 Hz/% RH	None	3.9% RH	8 s	[145]
MOF−hollow ball TiO_2_	0–97% RH	33.8 Hz/% RH	None	None	5 s	[146]
MOF−SnO_2_/CS	0–97% RH	43.1 Hz/% RH	None	3.05% RH	8 s	[147]

**Table 6:** Sensing properties of other MOF−based types of humidity sensors.

Materials	Types	Humidity Range	Response/ Sensitivity	Linearity (R^2^)	Response Time	Ref.
Cu−BTC	FPI	40 ppb–2000 ppm	0.4 mAU	None	10 s	[148]
GO/Co−MOF−74	LPFG	30–50% RH 50–90% RH	0.204 nm/% RH 0.16 dB/% RH	0.981 0.993	None	[149]
MOF−801/TiO_2_	PCF	20–90% RH	119 pm/% RH 1.34 dB/% RH	0.989	0.1 s	[150]
SiO_2_−HKUST−1	PCF	500–20,000 ppm	−29%	None	14 s	[151]
CDs−Co_3_O_4_	Microwave	5–99% RH	3.40 MHz/% RH 0.15 dB/% RH	0.993	5 s	[58]
Cu−BTC	SAW	3–14,800 ppm	0.23 ng/cm^2^	None	None	[152]
HKUST−1	colorimetric	1–5% RH	>100%	None	None	[153]

**Table 7:** Sensing properties of different MOF−based humidity sensors.

Materials	Type	Humidity Range	Response/ Sensitivity	Hysteresis	Linearity (R^2^)	Response Time	Ref.
LiCl@UiO−66−NH_2_	Impedance	11–95% RH	10^4^	4% RH	0.994	6 s	[82]
U−DPA−Zn	Impedance	11–97% RH	8070	1.8% RH	0.994	2 s	[84]
IL_15_−MOF−801	Impedance	0–35% RH	20.3	1% RH	None	0.4 s	[53]
PB derived Fe_2_O_3_	Impedance	11–95% RH	1568	0.59% RH	0.992	<2 s	[88]
Cu−BTC	Capacitive	20–100 ppm	1.13 pF/ppm	None	None	mins	[96]
MIL−96(Al)	Capacitive	0.7–90% RH	0.6 fF/% RH	None	None	None	[54]
MOF derived Co_3_O_4_	Resistive	10–95% RH	2730	2.6% RH	0.993	1 s	[55]
HIB−Cu	Resistive	1–1000 ppm	8	None	None	21 s	[107]
Ni−HAB	Resistive	20–90% RH	>10	None	None	4.9 s	[111]
TOCNF−MOF	Fluorescent	0–100% RH	Quenched	None	None	None	[56]
Zn(hpi2cf)(DMF) (H_2_O)	Fluorescent	0–1.3% *v*/*v*	Increased	None	0.935	None	[131]
[Mg(H_2_dhtp)(H_2_O)_2_]· DMAc	Fluorescent	0–5% *v*/*v*	Increased	None	None	None	[132]
[Co_2_(DPNDI)(2,6−NDC)_2_]⋅7(DMF)}*_n_*	Fluorescent	0–3% *v*/*v*	Increased	None	None	None	[134]
CNT−HKUST−1	QCM	5–75% RH	141 Hz/% RH	<5% RH	None	4.2 min	[144]
MOF−ZnCo_2_O_4_/ PPy	QCM	0–97% RH	58.4 Hz/% RH	3.9% RH	None	8 s	[145]
Cu−BTC	FPI	40 ppb–2000 ppm	0.4 mAU	None	None	10 s	[148]
MOF−801/TiO_2_	PCF	20–90% RH	119 pm/% RH	None	0.989	0.1 s	[150]
Cu−BTC	SAW	3–14,800 ppm	0.23 ng/cm^2^	None	None	None	[152]
HKUST−1	Colorimetric	1–5% RH	>100%	None	None	None	[153]

## Data Availability

Not applicable.
